# Effect of Ultrafine Fly Ash and Water Glass Content on the Performance of Phosphorus Slag-Based Geopolymer

**DOI:** 10.3390/ma15155395

**Published:** 2022-08-05

**Authors:** Jin Yang, Xiaolei Yu, Xingyang He, Ying Su, Jingyi Zeng, Fei Dai, Shiyu Guan

**Affiliations:** 1 School of Civil Engineering, Architecture and Environment, Hubei University of Technology, Wuhan 430068, China; 2 Building Waterproof Engineering and Technology Research Center, Hubei University of Technology, Wuhan 430068, China; 3 China Construction Third Bureau First Engineering Co., Ltd., Wuhan 430040, China

**Keywords:** phosphorus slag, geopolymer, fly ash, alkali-activated materials

## Abstract

Phosphorus slag (PS), an industrial waste slag, has been used in geopolymers because it is rich in silicon and calcium. The poor performance of phosphorus slag-based geopolymer is due to its aluminum deficiency. In this work, low-calcium fly ash, treated by a wet-grinding process, named wet-grinding ultrafine fly ash (WUFA) was used as an Al supplement to replace some of the phosphorus slag, and the wet-grinding, ultrafine fly ash-phosphorus slag (WUFA-PS)-based geopolymer was prepared. The effects of the substitution amount of WUFA and the activator dosage on the hydration properties, mechanical properties, pore structure and SEM of the WUFA-PS geopolymer were discussed in detail. The results indicate that WUFA and more activators contribute to the Al and high alkalinity environment, which positively induces the production of more geopolymer gels, thus releasing more heat and optimizing the pore structure of the matrix. The compressive strength increased by up to 28.1%. The enhanced performance of the WUFA-PS-based geopolymer may also arise from the filling effect and activity improvement of WUFA. This study has proved the feasibility of preparing a geopolymer by blending wet-grinding ultrafine fly ash and phosphorus slag and has provided references for the ratio and performance evaluation of WUFA-PS-based geopolymer concrete.

## 1. Introduction

Phosphorus slag (PS) is an industrial solid waste from the production of yellow phosphorus. The annual emission of phosphorus slag exceeds 8 million tons in China, and the amount of phosphorus slag stockpiles was approximately 100 million tons by 2020, and its massive stockpiles have caused serious pollution to the environment; therefore, there is an urgent need to find an effective resource-based disposal method for phosphorus slag [1,2]. The current application of phosphorus slag is mainly in the cement and concrete industry, where phosphorus slag can be used to prepare mineral admixtures and aggregates for mortar or concrete after treatment [2,3,4]. However, in China, the comprehensive utilization rate of phosphorus slag is still less than 50%, and it has been the research direction of many scholars to develop a utilization method for phosphorus slag and use it efficiently [5].

Geopolymer is an environmentally friendly, inorganic cementitious material, with a three-dimensional network structure, mainly formed by the depolymerization and repolymerization of silicon–oxygen and aluminum–oxygen tetrahedra. It has received much attention among various countries’ researchers because of its low energy consumption, low carbon emission, excellent performance, and the ability to use a large amount of industrial solid wastes [6,7,8,9]. The preparation of geopolymer with phosphorus slag as a precursor is a new sustainable development method, which can effectively utilize the metal elements in phosphorus slag, and there have been some fundamental studies on phosphorus slag-based alkali-activated geopolymers [10,11,12,13]. Maghsoodloorad et al. [14] explored the effect of different curing methods on the mechanical properties of alkali-activated phosphorus slag cementitious materials, indicating that the mechanical properties of phosphorus slag-based geopolymer are acceptable, although phosphorus slag lacks the aluminum phase. Liu et al. [10,15] and Mehdizadeh et al. [16] investigated, in some detail, the rheological properties and microstructure of fresh slurries of alkali-activated phosphorus slag-based geopolymer. In addition, the reaction kinetics of alkali-activated phosphorus slag-based geopolymer was also scrutinized and kinetic models were constructed by Liu et al. [17]. However, phosphorus slag is a material lacking the aluminum phase, which is an important element in the composition of geopolymer gels. Therefore, the mechanical properties of phosphorus slag-based geopolymer are unsatisfactory, and they are usually mixed with some aluminum-containing materials, as the raw material of geopolymers [18,19]. Class F fly ash is a low-calcium, high-alumina industrial by-product from thermal power plants, which is not suitable as a supplementary cementitious material due to its low volcanic ash activity but is commonly used as the raw material of geopolymers [20,21,22]. Low-calcium fly ash can supplement the aluminum component of phosphorus slag, while the calcium of phosphorus slag compensates for the shortcomings of fly ash. It was found that fly ash activity is directly related to their particle size [23,24,25].

In this paper, based on the wet-grinding process developed by our team, ultra-high activity, wet-grinding ultrafine fly ash (WUFA) was prepared [26,27,28]. The alkali-activated WUFA-PS-based geopolymer was prepared by using phosphorus slag and wet-grinding ultrafine fly ash as the main raw materials, and sodium silicate as the activator. The effects of the ultrafine fly ash volume and the water glass dosage on its mechanical properties, heat of hydration, autogenous shrinkage, hydration products, microstructure and resistance to chloride ion penetration were studied. The discussion in this paper was expected to provide a theoretical reference for the proportional design and preparation of a high-performance wet-grinding fly ash-phosphorus slag-based geopolymer.

## 2. Materials and Methods

### 2.1. Raw Materials

The phosphorus slag (PS) was obtained from Hubei Xingfa Chemicals Group Co., Ltd. (Yichang, China), with a median particle size of 45.3 μm. The raw fly ash (RFA) was low-calcium fly ash, produced by the Hanchuan Power Plant in Hubei province, with a median particle size of 19.7 μm. The main chemical composition of PS and RFA are shown in Table 1. The median particle size of wet-grinding ultrafine fly ash (WUFA) was 2.3 μm, and the detailed preparation process was described in [29,30,31]. Sodium silicate (SS) has an original modulus (factory modulus at purchase) of 3.3 and solids content of 40%. The modulus of SS was blended to 1.2, using sodium hydroxide. The fine aggregate was natural river sand with a fineness modulus of 2.82 and bulk density of 1460 kg/m^3^.

### 2.2. Mixture Proportions

The water–binder ratio of all groups was 0.5, and the binder–sand ratio was 1:2. PS, WUFA and river sand were first blended in the mixer, according to the experimental proportion in Table 2, and then SS and water were poured into the mixer, in turn, for stirring, and then the mortar was poured into the molds. The water content of SS and WUFA were 60% and 51%, respectively. Water in SS and WUFA was calculated and considered as blending water in the geopolymers. The molds were firstly placed in a standard curing room at 20 ± 2 °C and 95 ± 2% RH for 4 h, and then placed in a steam curing chamber at 60 °C and 95 ± 2% RH for 15 h. Finally, the specimens were demolded and placed in the standard curing room for further curing. Samples for DTA-TG, XRD and MIP were obtained from the core of the paste specimen, without river sand. To simplify the experiments, only PS85 (PS85-15, PS85-20 and PS85-25) and PS100-25 were chosen for detailed testing of autogenous shrinkage, XRD, MIP and SEM of WUFA-PS and PS-based geopolymers.

### 2.3. Testing Methods

#### 2.3.1. Compressive Strength Test

YAW-300C type pressure tester (Sanyu, Cangzhou, China) was used to test the compressive strength of the specimens, with a range of 0~300 kN. The loading rate was 2400 N/s, according to GB/T-17671 [32]; the size of the tested block was 40 × 40 × 40 mm^3^, and three specimens in the same group were tested at each age; and the average of the three strengths was taken as the final result. The tests were conducted at 3 days, 7 days and 28 days after the blocks were formed.

#### 2.3.2. Heat of Hydration Test

TAM Air isothermal calorimetry was applied to study the early hydration process of WUFA-PS geopolymer. Heat flow and cumulative release heat of hydration were recorded for each group of samples over 72 h, and the temperature of the test environment was kept at 20 °C. To ensure the accuracy of the test results, the experimental material was left in the test environment for a period of time before being placed in the test bottles for measurement.

#### 2.3.3. Autogenous Shrinkage Test

The geopolymer mortar was made into 40 mm × 40 mm × 160 mm specimens, cured for 24 h under standard curing conditions, the surface water was wiped and it was wrapped tightly with polyethylene plastic film to isolate the specimens from the outside environment. The specimens were placed on the automatic concrete shrinkage and expansion instrument, with the ambient temperature controlled at 20 ± 2 °C. The length change in the specimens was measured by the electronic displacement probe and the data were automatically collected by the microcomputer equipped with the instrument.

#### 2.3.4. Thermogravimetric Analysis (TG)

The STA 449 F5 Jupiter thermogravimetric analyzer, Hitachi, Berlin, Germany, was used to measure the mass change in the samples during the temperature increase. Samples with particle size less than 80 μm were dried under vacuum for 24 h before testing. 40 mg powdered samples were placed in a crucible at a heating rate of 10 °C/min, from 40 °C to 350 °C, with N_2_ flow rate of 50 mL/min.

#### 2.3.5. X-ray Diffraction (XRD)

A D8-Advance X-ray diffractometer from Bruker, Karlsruhe, Germany, was used to explore the crystal phase in the samples. The dried sample particle size was less than 60 μm, the scanning range was 10°~60°, and the scanning speed was 10°/min.

#### 2.3.6. Mercury Intrusion Porosimetry (MIP)

The pore structure of the specimens was analyzed using Poremaster-60 Mercury Injection Apparatus from Quantachrome, Boynton Beach, FL, USA. The sample with particle size of approximately 4–5 mm was oven dried for 24 h at 50 °C to ensure the pores were sufficiently dry. The contact angle between the paste and the mercury was chosen as 130° [33]. The pore size distribution was acquired via tested data.

#### 2.3.7. Chloride Penetration Resistance Test

According to ASTM C1202, the mortars were made into columnar specimens with a size of 50 mm × φ84 mm. After curing for 27 days, the blocks were soaked in clean tap water for 24 h and were fixed in the electrical flux tester, the cathode side was 3 wt % NaCl solution, while the anode side was 0.3 M NaOH solution. At room temperature, a voltage of 60 V was applied to the specimen, and the current was recorded every 30 min for 6 h. The electric flux value was the average of three blocks.

#### 2.3.8. SEM Test

The FEI-Quanta FEG 450 SEM was applied to observe the morphology of PS and WUFA-PS geopolymer hardened pastes, which had been cured for 28 days. It was operated in high vacuum mode, with an acceleration voltage of 15 kV and a working distance of approximately 10 mm. The samples were dried in an oven at 50 °C for 24 h before the test.

## 3. Results and Discussion

### 3.1. Heat of Hydration

**Figure 1:**
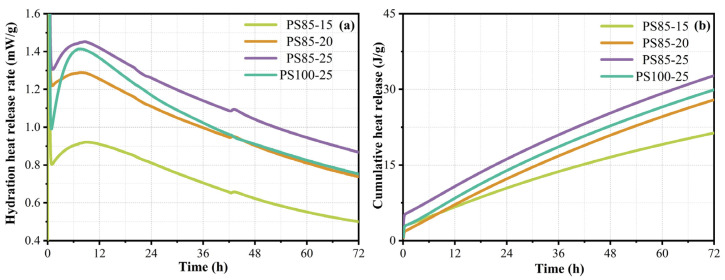
Hydration heat curves of WUFA-PS and PS-based geopolymers: (**a**) rate of heat release and (**b**) cumulative heat release.

The heat flow and cumulative heat release curves of the WUFA-PS-based geopolymers are shown in Figure 1. The exothermic reaction of the geopolymers was a rapid process, and, at the moment of contact with the activator, the crystal structure in the fly ash and phosphorus slag depolymerized in an alkaline environment; consequently, a large number of Ca-O bonds, Si-O bonds and the Al-O bonds were broken, releasing heat. This process cannot be completely detected, only the right half of the peak was shown in the curves. Furthermore, the peak of the main release of heat was located at 11 h, which was attributed to the condensation generation of the geopolymer gels.

It can be seen from Figure 1a,b, that the heat release rate and the total heat release of the WUFA-PS-based geopolymers increased with the amount of activator dosing, when the PS was constant. It has been demonstrated that high alkalinity can promote the breaking of Ca-O, Si-O and Al-O bonds in raw materials and increase the degree of reaction in raw materials, which is consistent with the patterns exhibited by the compressive strength. On the other hand, PS85-25 and PS100-25 were picked out to evaluate the role of WUFA. The heat release rate and the total heat release of PS85-25 were significantly higher than that of PS100-25, which was related to the WUFA providing Al for the formation of N-A-S-H and C-A-S-H. The activity of FA was elevated after the wet-grinding treatment, as shown in Figure 1a, near 1 h. The rate of heat release of PS85-25 was nearly 30% higher than that of PS100-25, which was attributed to the easier dissolution of Al and Si in the WUFA.

### 3.2. Compressive Strength

**Figure 2:**
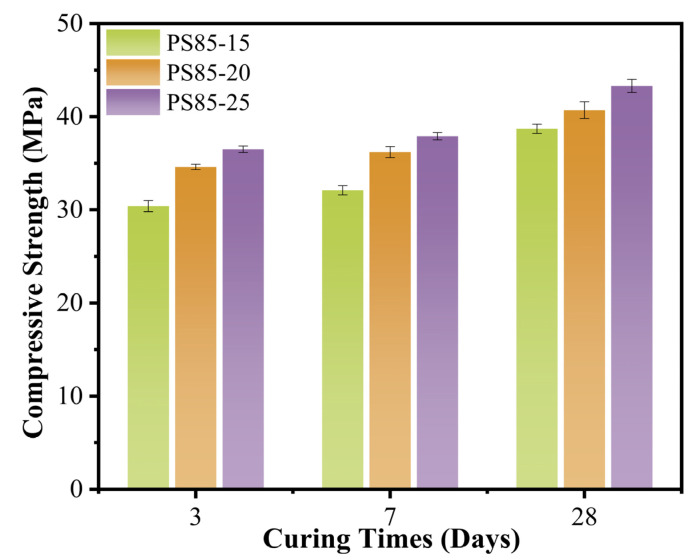
The compressive strength of WUFA-PS geopolymer mortars with different activator admixtures.

**Figure 3:**
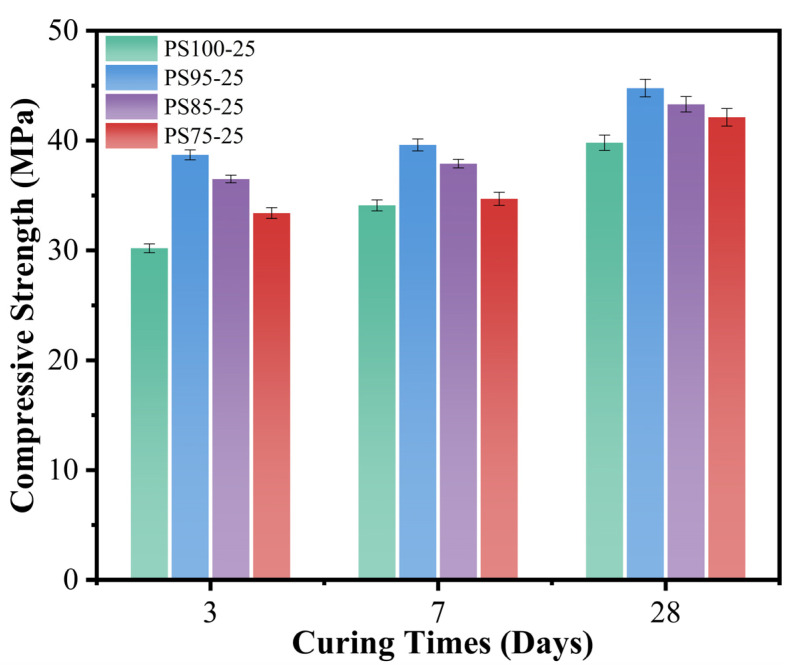
The compressive strength of WUFA-PS and PS-based geopolymer mortars with different WUFA substitution amounts.

Figure 2 and Figure 3 showed the compressive strength of the WUFA-PS-based geopolymer mortars at 3 days, 7 days and 28 days. It can be clearly seen from Figure 2 that the compressive strength of the WUFA-PS-based geopolymers increased gradually with the increasing number of activators. The compressive strength of PS85-20 and PS85-25 was increased by 13.8% and 20.1%, respectively, compared to PS85-15 at the age of 3 days. It is well known that the amount of gel products is positively correlated with the compressive strength in geopolymer materials, and the amount of activator used was directly related to the alkalinity of the system. High alkalinity provided a better environment for the dissolution of Ca, Si and Al, and for condensation reactions in the phosphorus slag and fly ash, promoting more geopolymer gels, such as C-(A)-S-H and N-A-S-(H) [34,35].

Another significant trend can be seen in Figure 3, where the compressive strength of the geopolymer mortars were all improved when WUFA replaced some of the PS, at the same amount of activators. It is worth noting that the compressive strength of PS95-25 reached 38.7 MPa at 3 days, which increased by 28.1% compared to PS100-25. The reasons can be attributed to the following three aspects: (1) WUFA supplied Al to the geopolymer mortars and provided decisive conditions for the generation of N-A-S-H and C-A-S-H; (2) wet-grinding disposal pre-destroyed the spherical phase structure of FA, and Al dissolved more easily from the crystalline phase; and (3) ultrafine WUFA served as a filler to fill the rough pores in the geopolymer matrix [36]. It cannot be ignored that the compressive strength of the WUFA-PS geopolymer mortars were slowly decreased with the increase in WUFA to PS substitution, which was related to the Si/Al in the system. With the incorporation of WUFA (the main source of Al), the Si/Al ratio decreased and the structure was transformed from amorphous and glassy, to brittle zeolite A and/or sodalite [37,38].

### 3.3. Autogenous Shrinkage

The early shrinkage of cementitious materials is a phenomenon caused by a combination of chemical and physical factors and is closely related to the volume shrinkage of the chemical reaction of the cementitious material and the drying shrinkage caused by autogenous drying [39]. The autogenous shrinkage of the geopolymers was positively correlated with the amount of geopolymer gels produced under test conditions, without moisture exchange with the outer environment.

**Figure 4:**
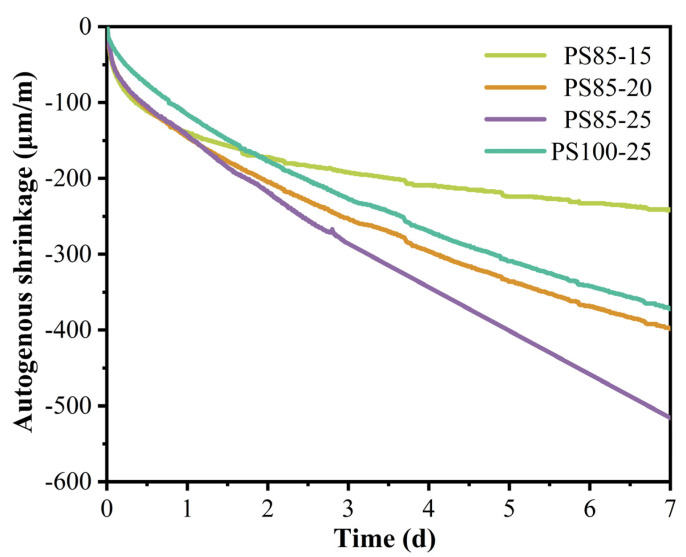
The autogenous shrinkage of WUFA-PS and PS-based geopolymer mortars with different amounts of activators.

The autogenous shrinkage of the WUFA-PS-based geopolymer mortars are shown in Figure 4. It is obvious that there was a significant increase in the autogenous shrinkage of the geopolymer mortars as the amount of sodium silicate incorporation increased. The more gel products there were, the more water was consumed and the drier the mortar matrix was. This was another validation that the addition of more activators (20% and 25%) encouraged the production of more gel products.

In terms of whether WUFA was added or not, PS85 and PS100-25 were selected and compared. It can be noticed that the autogenous shrinkage of PS85-25 was much greater than that of PS100-25, which is further evidence that the Al introduced into the system by the WUFA promoted the generation of geopolymer gels. It is necessary to point out that the autogenous shrinkage of the WUFA-PS-based geopolymer mortars was greater than that of the PS-based in all cases at the early stage (1 day), even with a lower content of activators. This corroborated the previous section on the enhancement of FA activity by wet-grinding disposal.

### 3.4. Chloride Penetration Resistance

The amount of electric flux could represent the resistance of the specimen to chloride ion penetration: the larger the electric flux, the lower resistance of the specimen to chloride ion. The electric flux value was closely related to the pore structure and the degree of compactness of the geopolymer cementitious material. In general, the disconnected pore network structure and the dense microstructure both significantly reduced the electric flux of the geopolymer. The concentration of Na^+^ and OH^−^ in the pore solution of alkali-activated geopolymer materials was higher than the cement-based materials, resulting in a low concentration of Cl^−^, so the electric flux of the alkali-activated geopolymers was universally lower [40].

**Figure 5:**
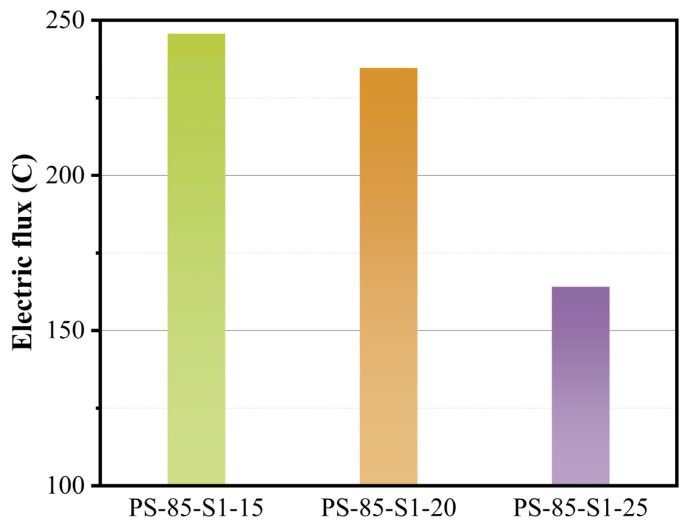
The electric fluxes of WUFA-PS geopolymer mortars with different activator dosages.

**Figure 6:**
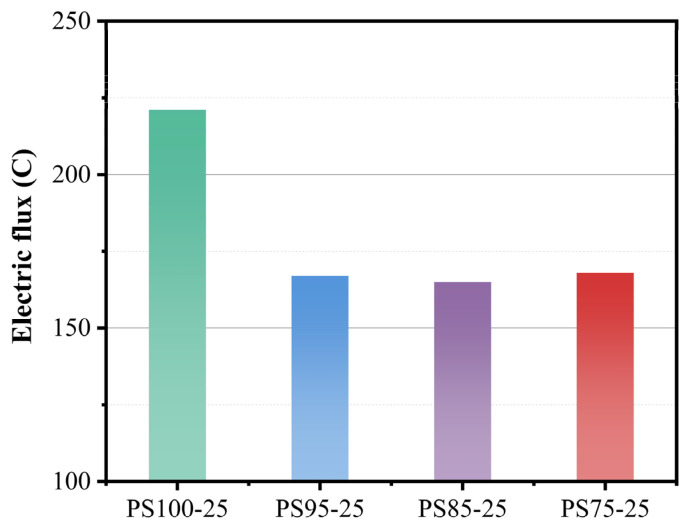
The electric fluxes of WUFA-PS with different WUFA substitutions and PS-based geopolymer mortars.

As shown in Figure 5, the electric flux of the WUFA-PS-based geopolymer mortars gradually decreased with the increase in the activator content. This result was highly consistent with the strength results, and the reason was similar to the strength analysis, above, confirming that more activators promote more gel products. The electric fluxes of the WUFA-PS geopolymer mortars, with different WUFA replacements, are shown in Figure 6. It is evident that the WUFA-PS geopolymers, generally, have a better resistance to chloride ion penetration than the PS-based geopolymers. In addition to the activity improvement, the ultrafine fly ash particles were effectively filled in the geopolymer cementitious structure, which reduced the connectivity of the pore structure. Furthermore, compared to C-(A)-S-H, the N-A-S-(H) generated by the WUFA and PS under alkali activation had stronger resistance to corrosion and could adsorb chloride ions without easy disintegration, thus enhancing the chloride ion permeability of the specimens [41,42].

### 3.5. TG

**Figure 7:**
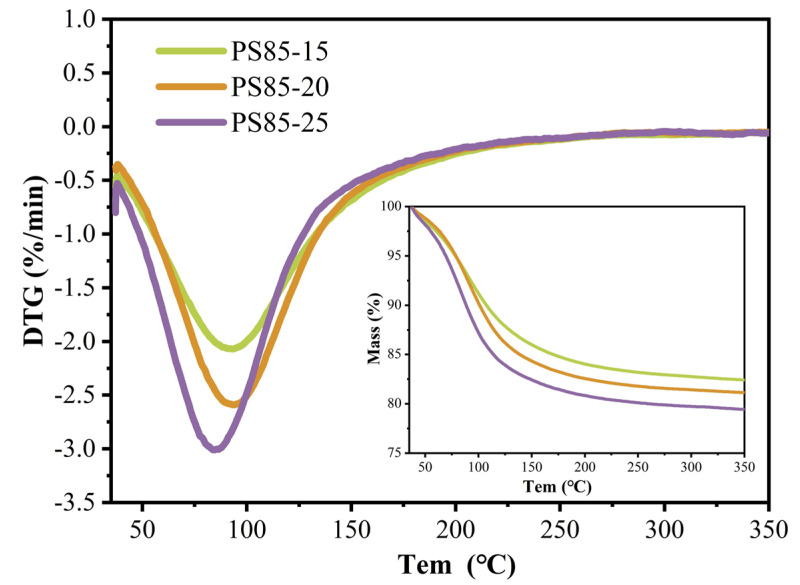
TG-DTG curves of WUFA-PS-based geopolymers with different activator contents at 28 days.

Figure 7 presents the thermal loss curves of the WUFA-PS-based geopolymers, with different activator doping. Thermogravimetric analysis is a common approach, used to analyze the hydration gel products of cementitious materials quantitatively, and this method has also been adapted to the quantitative detection of geopolymer gels [43,44]. According to previous studies, the mass loss at 50–200 °C was caused by the water loss of the geopolymer gels, which was proportional to the number of gels [44,45]. In contrast to cement-based materials, no Ca(OH)_2_ was produced during the formation of the geopolymer gels, so the thermal loss of calcium hydroxide was not considered.

What was clear in our study, was that the higher the activator content, the higher the weight loss of the gel products, and that the elevated alkalinity and Si supplementation drove the production of more geopolymer gels (N-A-S-H and C-A-S-H),which also demonstrated that the increase in strength in Section 3.2 was due directly to the increased number of geopolymer gels in the geopolymer mortar. Similarly, the previous arguments related to heat of hydration, autogenous shrinkage and durability are confirmed here.

**Figure 8:**
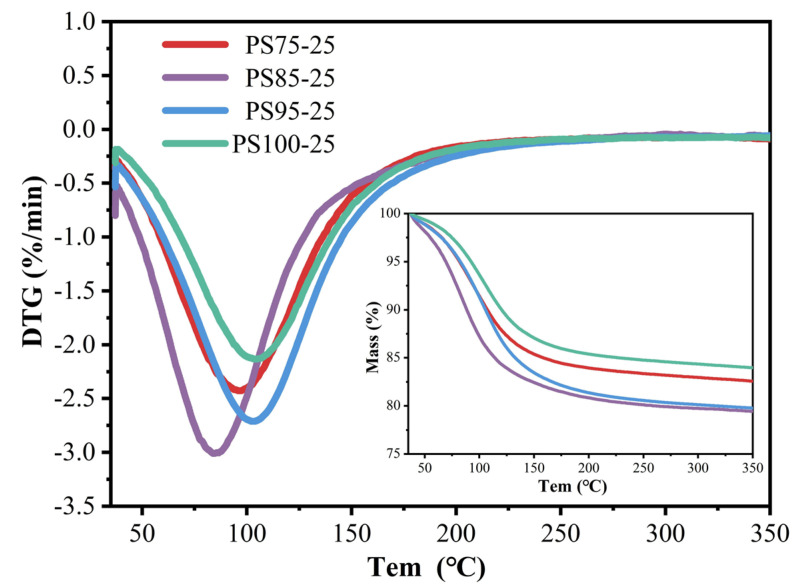
TG-DTG curves of WUFA-PS with different WUFA substitutions and PS-based geopolymer at 28 days.

As shown in Figure 8, the weight loss of the WUFA-PS geopolymers with different WUFA incorporation were higher than that of the single PS-based geopolymer in the geopolymer gels’ thermogravimetric loss interval. The supplementary Al of WUFA for the cementation system and its own activity enhancement, synergistically enhanced the yield of geopolymer gels. The results of the thermogravimetric loss were consistent with the previous findings in this paper, and it was further validated that the enhanced mechanical properties and resistance to chloride ion penetration of the PS geopolymer, by the added WUFA, were related to the increase in gel products. It is worth noting that the amount of thermogravimetric loss of PS75-25 was less than that of PS85-25 and PS95-25, which also corresponds to its lower compressive strength, compared to PS85-25 and PS95-25 in Section 3.2. What can be conjectured, is that Si/Al affected the formation of geopolymer gels.

### 3.6. XRD

**Figure 9:**
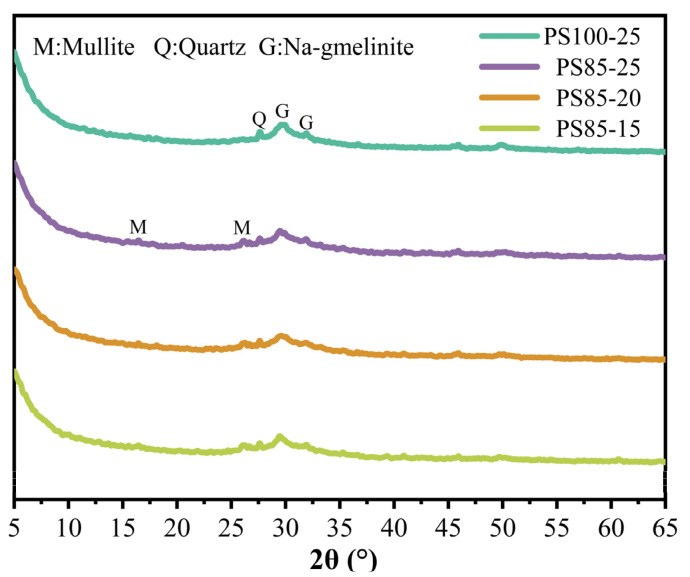
XRD patterns of WUFA-PS and PS-based geopolymers at 28 days.

The XRD patterns of WUFA-PS and PS-based geopolymers were shown in Figure 9. Firstly, the quartz phase was detected in all the geopolymers that originated from the raw materials, PS and WUFA. Only the WUFA-PS geopolymer contained the mullite phase, due to the existence of mullite in WUFA [46]. Notably, the hump near 30° 2θ corresponded to the gmelinite phase: a sodium-containing aluminosilicate mineral, indicating the formation of geopolymer crystal products [18,38]. Since the heat curing time was short, most of the condensation products of the raw materials were amorphous gels, so XRD was unable to detect all of the geopolymer products, and only some of the crystal products that coexisted with the gels were detected [47,48].

### 3.7. Pore Structure

**Figure 10:**
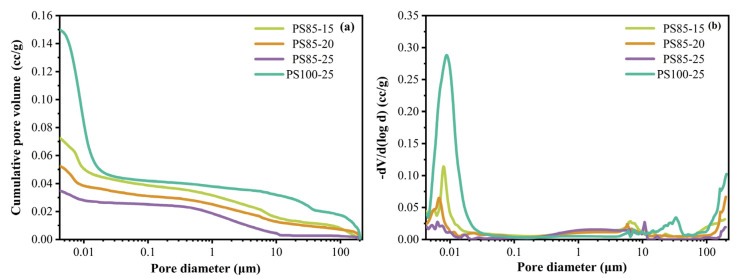
Pore size distribution of WUFA-PS and PS-based geopolymers at 28 days: (**a**) cumulative curves and (**b**) differential curves.

The pore size distribution of the WUFA-PS and PS-based geopolymers at 28 days are displayed in Figure 10. PS85 and PS100-25 were chosen when comparing the amount of WUFA substitution. As seen in Figure 10a, the cumulative pore volume of the samples continuously decreased with the addition of more activators, which was attributed to the fact that the increase in activators prompted more geopolymer gels to be produced, filling the pore structure and making the geopolymer matrix even denser. Moreover, the cumulative pore volume of the WUFA-PS-based geopolymers were much lower than that of a single phosphorus slag-based geopolymer, which further confirms the filling effect and activity enhancement proposed in the Section 3.2 and Section 3.4. In the same way, it can be seen from Figure 10b that both the enhanced activator and the incorporation of WUFA contributed to the leftward shift of the most countable pore size of the geopolymer samples, directly demonstrating that the larger pores of the geopolymer matrix were filled by the increased gel products and ultrafine fly ash particles.

In order to further analyze the pore distribution of samples, the pores in the WUFA-PS and PS-based geopolymer samples were divided and counted according to their sizes. Referring to the pore division method of cement-based materials, the pore size ranges of less than 10 nm, 10–50 nm and 50–100 nm, and greater than 100 nm, were called gel pores, fine capillary pores, medium capillary pores and large capillary pores, respectively [49,50,51].

**Figure 11:**
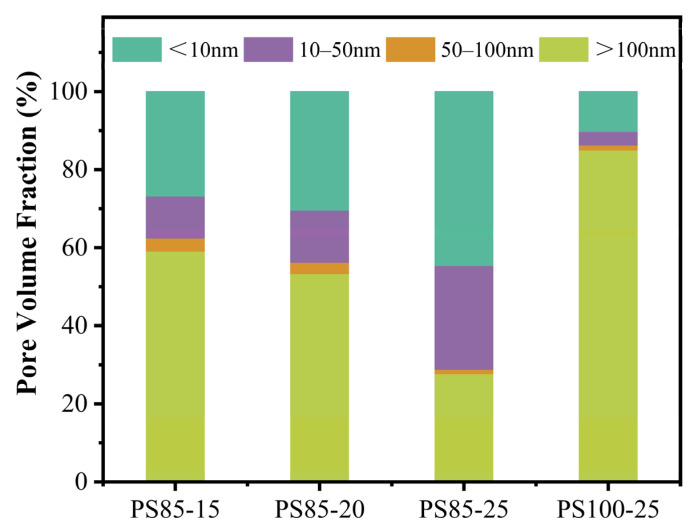
Pore volume fraction of WUFA-PS and PS-based geopolymers at 28 days.

The pore volume fraction results are shown in Figure 11. It can be seen intuitively that with the incorporation of more and more activators, the proportion of large capillary pores in the WUFA-PS-based geopolymers became smaller and smaller, while the proportion of gel pores and fine capillary pores continued to increase. The gel pores are generally pores of gel structure. The increase in the gel pore ratio confirms the increase in gel products. In addition, some of the large capillary pores were filled by condensation products, making them into fine capillary pores. Last but not least, replacing some of the PS with WUFA, as a raw material for geopolymers, effectively optimized the pore structure of a single PS-based geopolymer. The ratio of the large capillary pores of PS85-25 was much smaller than that of PS100-25, while its proportion of gel pores and fine capillary pores was several times that of PS100-25. The pore structure of PS85-15 was still far better, even if the amount of the activator was less than that of PS100-25. On the one hand, the improved activity of WUFA and the provision of Al to the geopolymer precursor required for the condensation reaction prompted more N-A-S-(H) production, which was the original reason for the increased percentage of gel pores. In addition, the median particle size of WUFA was only 2.31 μm, which enabled it to fill the larger capillary pores in the geopolymer and achieve a closer packing effect, reflecting a significant decrease in the fraction of large capillary pores. It is well known that the porosity of cementitious materials has a straightforward correlation with strength, and the pore structure results in this section coincided with the results of strength in the previous section, further confirming the positive effect of WUFA on the mechanical properties of PS geopolymers.

### 3.8. SEM

**Figure 12:**
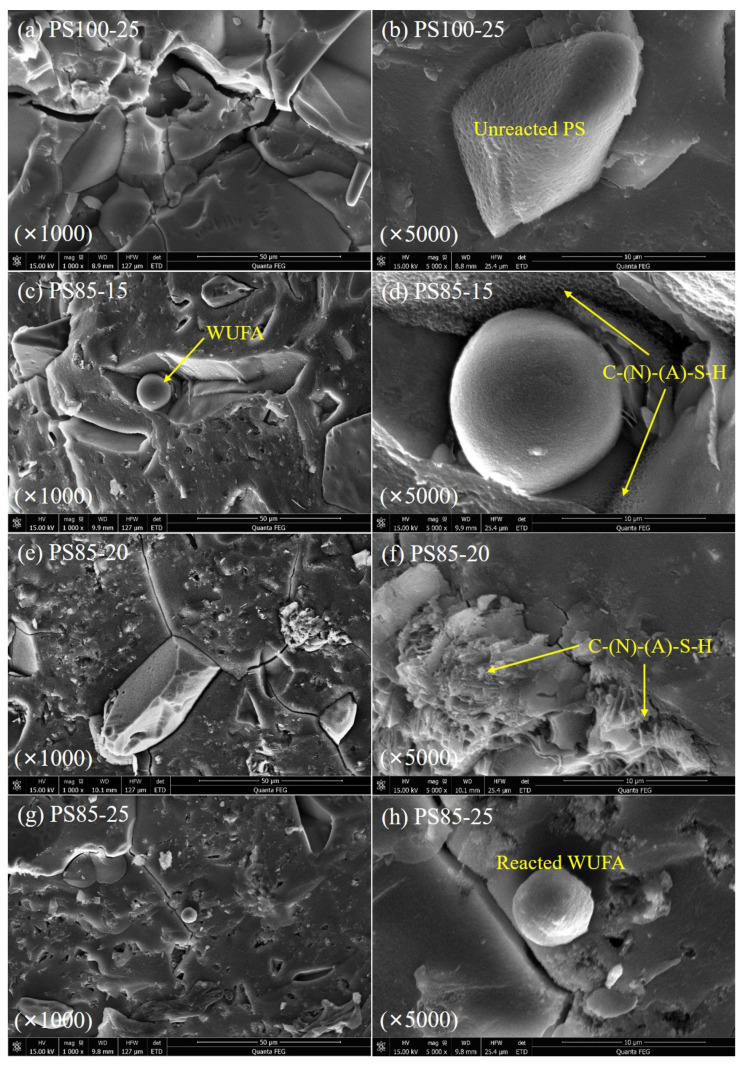
SEM images of PS (**a**,**b**) and WUFA-PS (**c**–**h**)-based geopolymers at 28 days.

To further study the effect of WUFA on PS geopolymers and of the activator dose on the microstructure of WUFA-PS geopolymers, the microscopic morphology of the geopolymer-hardened pastes was taken by scanning electron microscopy, and the photographs are presented in Figure 12. It can be seen in Figure 12a that there are many in PS100-25s, and the overall matrix was relatively loose. The unreacted PS can be seen in Figure 12b, indicating the low level of hydration of the PS-based geopolymer. In contrast, after the WUFA was introduced, the matrix of the geopolymer was denser, with fewer sparse structures and cracks, as can be visualized in Figure 12c,e,g. Notably, that the WUFA filled in the pores of the geopolymer matrix can be observed in Figure 12c, where the filling effect of the WUFA was confirmed more clearly. Meanwhile, from Figure 12d, one can notice that the geopolymer gels, which are attached to the inner wall of the pore filled by WUFA, were probably N-A-S-H and the C-A-S-H, because WUFA may contribute Al to the surrounding areas, indicating the active role performed by WUFA in the geopolymer gel generation process. On the other hand, the matrix of the geopolymer became denser as the activator dose increased, which correlated with the amount of hydrated gel. Dense gel products can be observed in Figure 12f. This, to some extent, suggests that higher doses of activator can induce more geopolymer gels to be produced. In addition to this, the reacted WUFA was detected in PS85-25, as shown in Figure 12h. In the system with high activator content, fine FA particles reacted, and the surface of the spherical particles had a rough texture due to the formation of gels.

## 4. Conclusions

In the present work, wet-grinding ultrafine fly ash was incorporated into the phosphorus slag-based geopolymer, and the effect of the activator dosage on the hydration and mechanical properties of the WUFA-PS-based geopolymer was discussed. The following conclusions can be drawn:

1. WUFA promoted the geopolymers to release more heat in the reaction, which is attributed to the Al in the WUFA being supplemented into the geopolymer and being used to generate more N-A-S-(H) gels. The increase in the activator content elevated the alkalinity of the reaction environment, which led, in turn, to a more intense condensation reaction.
2. The compressive strength of the WUFA-PS-based geopolymer was significantly improved, compared to that of a single PS, possibly due to the supplementation of the missing elements in PS and the filling effect of WUFA. The compressive strength of PS85-25 was increased by 20.1%, compared to PS85-15, at the age of 3 days. Similarly, the improvement in the ratio of the activator provided more convenient conditions for the enhancement of mechanical properties.
3. The autogenous shrinkage and thermogravimetric test confirmed that WUFA caused more gel products in the WUFA-PS-based geopolymer than in the PS, which equally exacerbated the autogenous shrinkage of the specimens.
4. WUFA and the addition of more activators decreased the porosity of the geopolymers; filled a number of large capillary pores, making them fine capillary pores; and prompted the percentage of gel pores to keep rising. The proportion of gel pores in PS85-25 improved by 34.4%, compared to PS100-25, while the proportion of large capillaries decreased by 57.3%. In addition, the connectivity of the pores was reduced, as shown by the decrease in the value of the electric flux with the addition of WUFA and more activators.

## Figures and Tables

**Table 1:** Chemical composition of phosphorus slag and fly ash (%).

	CaO	Fe_2_O_3_	SiO_2_	MgO	Al_2_O_3_	TiO_2_	P_2_O_5_	SO_3_	F	LOI
PS	38.8	1.5	41.3	1.6	5.9	0.4	3.5	2.3	2.1	2.6
RFA	5.2	3.6	44.8	0.6	39.2	1.4	-	1.5	-	3.4

**Table 2:** Mix proportion of WUFA-PS and PS-based geopolymers.

Sample	PS (wt %)	WUFA (wt %)	SS (wt %)	River Sand (wt %)
PS100-25	100	0	25	200
PS95-25	95	5	25	200
PS85-25	85	15	25	200
PS75-25	75	25	25	200
PS85-20	85	15	20	200
PS85-15	85	15	15	200

## Data Availability

Not applicable.
